# Characteristics of Human OAS1 Isoform Proteins

**DOI:** 10.3390/v12020152

**Published:** 2020-01-29

**Authors:** Han Di, Husni Elbahesh, Margo A. Brinton

**Affiliations:** 1 Department of Biology, Georgia State University, Atlanta, GA 30303, USA; realer5@gmail.com (H.D.); Husni.Elbahesh@tiho-hannover.de (H.E.); 2 Research Center for Emerging Infections and Zoonosis (RIZ), University of Veterinary Medicine Hannover, 30559 Hannover, Germany

**Keywords:** human OAS1 isoforms, 2-5A, rRNA cleavage, Fibrillin1, supervillin

## Abstract

The human OAS1 (hOAS1) gene produces multiple possible isoforms due to alternative splicing events and sequence variation among individuals, some of which affect splicing. The unique C-terminal sequences of the hOAS1 isoforms could differentially affect synthetase activity, protein stability, protein partner interactions and/or cellular localization. Recombinant p41, p42, p44, p46, p48, p49 and p52 hOAS1 isoform proteins expressed in bacteria were each able to synthesize trimer and higher order 2′-5′ linked oligoadenylates in vitro in response to poly(I:C). The p42, p44, p46, p48 and p52 isoform proteins were each able to induce RNase-mediated rRNA cleavage in response to poly(I:C) when overexpressed in HEK293 cells. The expressed levels of the p42 and p46 isoform proteins were higher than those of the other isoforms, suggesting increased stability in mammalian cells. In a yeast two-hybrid screen, Fibrillin1 (FBN1) was identified as a binding partner for hOAS1 p42 isoform, and Supervillin (SVIL) as a binding partner for the p44 isoform. The p44-SVIL interaction was supported by co-immunoprecipitation data from mammalian cells. The data suggest that the unique C-terminal regions of hOAS1 isoforms may mediate the recruitment of different partners, alternative functional capacities and/or different cellular localization.

## 1. Introduction

Oligoadenylate synthetase (OAS) proteins are cellular, double-stranded RNA (dsRNA) sensors that function as part of the innate immune response to virus infections [1,2]. In the human genome, the OAS family consists of one copy each of the OAS1, OAS2, OAS3 and OASL genes. The OAS1 protein contains a single OAS domain, while the OAS2 and OAS3 proteins are composed of two and three OAS domains, respectively [3,4]. Human OASL contains an inactive OAS domain plus two domains of ubiquitin-like sequences [4,5,6]. Interaction with dsRNA or base-paired regions in a single-stranded RNA (ssRNA) activates OAS proteins to polymerize ATP into 2′-5′-linked oligoadenylates (2-5A). Trimer and higher order 2-5As bind to endoribonuclease L (RNase L), inducing its dimerization and activation. Activated RNase L cleaves viral and cellular ssRNAs, including 28S and 18S ribosomal RNA (rRNA) [1,7,8]. Although human OASL is catalytically inactive, upon dsRNA binding, it has an RNase L-independent antiviral activity that is mediated through enhancing RIG-I signaling [9,10].

Multiple alternatively spliced human OAS1 gene mRNAs have previously been amplified from human cells [11,12]. An A/G SNP (rs10774671) at the intron-5/exon-6 splice acceptor site alters hOAS1 pre-mRNA splicing [11,12,13,14]. The p46 isoform mRNA is predominantly produced by individuals with the G allele, while those with the A allele produce p48 and p52. 

Although all of these hOAS1 isoforms share the same 346 amino acid sequence in the N-terminal catalytic domain, they differ at the C-termini [15,16]. Individuals with one or two copies of the A allele were previously found to have lower OAS activity [12].

Antiviral activity for some hOAS1 isoforms, but not for others, was previously reported in response to infections with the mosquito-borne flaviviruses, West Nile virus (WNV) and Dengue virus, that are of public health importance. The p42 isoform was shown to be activated by interaction with a 5′ sequence of the WNV genome RNA, comprised of stem loops I, II and III [17]. A Dengue virus infection only induced RNase L activation in cells that were overexpressing the hOAS1 p42 and p46 isoforms, but not the p44, p48 or p52 isoforms [18].

In the present study, we analyzed the in vitro synthetase activities of seven bacterially-expressed recombinant hOAS1 isoform proteins, and all were found to synthesize 2-5A after incubation with poly(I:C). We then individually overexpressed five of the hOAS1 isoforms in HEK293 cells, and analyzed their ability to activate endogenous RNase L. Each of the hOAS1 isoforms activated RNase L in response to treatment of the cells with poly(I:C). The data indicate that all five of these isoforms are active synthetases, both in vitro and in mammalian cells. A yeast two-hybrid screen was utilized to identify novel binding partner candidates for the hOAS1 p44 and p42 isoforms. The data support a role for the unique C-terminal sequences of the different hOAS1 isoforms in recruiting unique partners that may facilitate additional functions and/or intracellular localization.

## 2. Materials and Methods

### 2.1. Cells

Human embryonic kidney HEK293 cells were cultured at 37 °C in a 5% CO_2_ atmosphere in Dulbecco’s Modified Eagle Medium (DMEM) supplemented with 1% L-glutamine, 5% fetal bovine serum (FBS) and 0.1% gentamicin. Human lung carcinoma A549 cells were cultured at 37 °C in a 5% CO_2_.atmosphere in F-12K nutrient media supplemented with 1% L-glutamine, 10% fetal bovine serum and 0.1% gentamicin.

### 2.2. Cloning and Expression of hOAS1 Isoform Proteins in Bacteria

Six plasmid DNAs each containing the cDNA for an individual human OAS1 isoform (p41, p42, p44, p46, p48 and p49) were provided by Dr. Andrey Perelygin (Georgia State University). A plasmid clone of the p52 cDNA was provided by Dr. Svetlana Scherbik (Georgia State University). The GenBank accession number for P49 is DQ914956.1, for p52 it is AY730627.1 and for p41 it is DQ823040.1. These cDNAs were previously amplified by reverse transcription--polymerase chain reaction (RT-PCR) amplification from RNA extracted from ATCC human fibroblasts CCL-110 or CCL-66 that had been treated with IFNβ for 24 h and cloned into a TOPO-TA vector. Individual clones were selected and sequenced to identify the different isoform cDNAs. Each hOAS1 isoform was then subcloned into the pET151-TOPO bacterial expression vector (Thermo Fisher Scientific, Waltham, MA, USA) with a 6 X His tag and a V5 epitope fused at the N-terminus.

The hOAS1 plasmid DNAs were transformed into One Shot TOP10 chemically competent *Escherichia coli* (*E. coli*) (Thermo Fisher Scientific, Waltham, MA, USA), re-isolated from colonies and sequenced. hOAS1 isoform proteins were expressed in transformed One Shot BL21-(DE3)-pLysS cells (Thermo Fisher Scientific, Waltham, MA, USA), grown in 125 mL of Luria-Bertani (LB) media containing 0.05% glucose and 100 μg/mL of carbenicillin (CRB). Expression was induced with 1 mM of isopropyl β-d-1-thiogalacto-pyranoside (IPTG) overnight at 16 °C. Cells were pelleted by centrifugation at 6000× *g* for 10 min at 4 °C. The cells were resuspended in 10 mL of 1 X Equilibration buffer [NaCl (300 mM), sodium phosphate (50 mM) and 1 X Complete Mini EDTA-Free Protease inhibitor cocktail (Roche, Indianapolis, IN, USA)] and frozen at −80 °C until use. CelLytic Express lysis powder (Sigma-Aldrich, St. Louis, MO, USA) was added to the thawed cell suspensions and incubated at 37 °C for 30 min with shaking. The cell lysates were clarified by centrifugation at 15,000× *g* at 4 °C for 10 min. The volume of the clarified supernatant was increased to 20 mL by addition of 1 X Equilibration buffer and transferred to a column containing 1 mL of TALON metal affinity nickel resin (Clontech, Mountain View, CA, USA). 

After washing the column with 1 X washing buffer [50 mM sodium phosphate (pH 7.4), 300 mM NaCl and 5 mM imidazole], the bound proteins were eluted with 5 mL of 1 X Elution buffer [50 mM sodium phosphate (pH 7.4), 300 mM NaCl and 150 mM imidazole]. The eluted protein fractions were combined, and the buffer was first exchanged with 1 X Storage buffer [20 mM Hepes-KOH (pH7.5), 50 mM KCl, 25 mM Mg(OAc)_2_, 7 mM β-ME, 0.03 mM ethylenediaminetetraacetic acid (EDTA), 0.25% glycerol and 1 X Complete Mini EDTA-Free Protease inhibitor cocktail (Roche, Basel, Switzerland)] and then concentrated using a Microcon-10 kDa Centrifugal Filter Unit (Millipore, Burlington, MA, USA). The partially purified proteins were aliquoted and stored at −80 °C.

### 2.3. In Vitro 2′-5′ OAS Activity Assay

Each adenosine triphosphate (ATP) polymerization reaction mixture (50 µL) contained an individual hOAS1 isoform protein (22 µL), α^32^p-ATP (15 µCi) and poly(I:C) (50 ng/µL) in 1 X Assay buffer [20 mM HEPES-KOH pH 7.5, 50 mM KCl, 25 mM Mg(OAC)_2_, 10 mM creatine phosphate, 1 U/µL creatine kinase, 5 mM ATP and 7 mM β-ME]. After incubation at 30 °C for 18 h, the reaction was stopped by the addition of 50 µL of Gel Loading Buffer II [95% formamide, 18 mM EDTA, 0.025% SDS, xylene cyanol and bromophenol blue (Ambion, Austin, TX, USA)]. An aliquot of the reaction (2 µL) was separated on a 20% polyacrylamide/Urea gel at 800 V for 3.5 h, and the production of radiolabeled 2-5A was detected by autoradiography.

### 2.4. Functional Analysis of hOAS1 Isoforms in Mammalian Cells

The hOAS1 p42, p44, p46, p48 and p52 isoform cDNAs were subcloned into the p3xFlag-CMV mammalian expression vector with a 3 X Flag tag fused at the N-terminus. HEK293 cells were seeded in a 6-well plate and grown to ~70% confluence before transfection with either empty vector DNA or with a hOAS1 isoform plasmid DNA using Lipofectamine LTX/PLUS reagent (Thermo Fisher Scientific, Waltham, MA, USA). At different times after the initial transfection, the cells were transfected with 0.5 µg of poly(I:C) for 6 h using Lipofectamine 2000 (Thermo Fisher Scientific, Waltham, MA, USA). Cell lysates were harvested in TRI reagent (Molecular Research Center. Inc., Cincinnati, OH, USA). Total intracellular RNA was extracted and purified according to the manufacturer’s protocol and then separated on a denaturing formaldehyde/3-(*N*-morpholino)propanesulfonic acid (MOPS) agarose gel. The RNA gel was stained with ethidium bromide and imaged under ultraviolet (UV) light. 

For protein detection by Western blotting, hOAS1 cDNA-transfected HEK293 cells were lysed by incubation with 1 X RIPA buffer [1 X phosphate-buffered saline, 1% Nonidet P-40, 0.5% sodium deoxycholate,0.1% sodium dodecyl sulfate (SDS) and 1 X Halt™ protease inhibitor cocktail (Thermo Fisher Scientific, Waltham, MA, USA)].

### 2.5. Yeast Two Hybrid Assay

A yeast two hybrid assay was performed using the Matchmaker Gold Yeast Two-Hybrid System following the manufacturer’s protocol (Clontech, Mountain View, CA, USA). Briefly, the cDNA sequences of the full length hOAS1-p42 and hOAS1-p44 isoforms were fused to the GAL4-BD domain in the pGBKT7 vector, and separately transformed into the Y2HGold yeast strain as the bait. A Y187 yeast strain containing a universal human cDNA library fused to the GAL4-AD domain in a pGADT7-RecAB vector (Clontech, Mountain View, CA, USA) was used as the prey. The Y2HGold and Y187 yeast strains were mated at 30 °C for 24 h and plated on double dropout (DDO, minus Trp and Leu) plates for selection of mated diploid cells. The selected diploid cells were then plated on triple dropout plates supplemented with Aureobasidin A (TDO/A, minus His, Trp and Leu) and quadruple dropout plates supplemented with Aureobasidin A (QDO/A, minus Ade, His, Trp and Leu) for the selection of positive clones that contained interacting prey and bait under increasingly stringent conditions. When the prey and bait interact, in addition to the expression of the missing components in the media, the AUR1-C gene is also expressed, which confers strong resistance to the otherwise highly toxic drug Aureobasidin A, so that the yeast can grow on media containing Aureobasidin A. 

The prey and bait plasmid constructs were extracted from yeast colonies that grew on QDO/A plates, and their cDNA inserts were sequenced. A basic local alignment search tool (BLAST) search was then performed to determine the identity of the putative binding peptide.

### 2.6. Yeast Co-Transformation

A plasmid expressing a putative binding peptide was transformed into the Y187 yeast strain. The transformed Y187 strain was then mated with the Y2HGold strain containing an hOAS1 bait at 30 °C for 24 h. The mated diploid cells were then plated on DDO, TDO/A and QDO/A plates to confirm the co-transformation, as well as the positive interaction, between the bait and the putative binding peptide.

### 2.7. In Vitro Transcription/Translation and Pull-Down Assay

The cDNAs of the full-length hOAS1 bait and the putative binding peptide were individually cloned into a pTNT expression vector (Promega, Madison, WI, USA) with a c-myc tag or an HA tag fused at the N-terminus, respectively. The resulting bait and prey constructs were then expressed in vitro in the presence of [^35^S]-methionine using a TnT-coupled transcription/translation system (Promega), according to the manufacture’s protocol. The in vitro translated bait and prey peptides were incubated together for 1 h at room temperature and then divided into three equal portions. One portion was stored at −20 °C and was used as a lysate sample, one portion was incubated with control agarose beads conjugated with a non-specific control IgG antibody, and one portion was incubated with agarose beads conjugated with either anti-c-myc or anti-HA antibody. After an overnight incubation at 4 °C with rotation, the beads were washed with 1 X Lysis buffer [Triton X-100 (1%), SDS (0.1%), NaCl (150 mM), Tris-HCl (50 mM) and Halt™ protease and phosphatase inhibitor cocktail (Thermo Fisher Scientific, Waltham, MA, USA)]. The washed beads were suspended in 2 X sample buffer [(SDS (20%), glycerol (25%), Tris-HCl (0.5 M), bromophenol blue (0.5%) and β-mercaptoethanol (5%)] and boiled for 5 min. The pulled-down protein complexes and the original lysate sample were separated by 10% sodium dodecyl sulfate-polyacrylamide gel electrophoresis (SDS-PAGE). The gels were first incubated in a fixing solution (10% acetic acid and 30% methanol), then in Autofluor (National Diagnostics, Atlanta, GA, USA) and finally in anti-cracking buffer (7% acetic acid, 7% methanol and 1% glycerol). The gels were dried onto 3 mm chromatography paper (Whatman, Cleves, OH, USA) and autoradiographed.

### 2.8. Mammalian Cell Co-Immunoprecipitation

A549 cells were seeded in a 10-cm plate and transiently transfected with the p3xFlag-CMV-OAS1-p44 construct. At 48 h after transfection, cell lysates were harvested in 1 X Lysis buffer [Triton X-100 (1%), SDS (0.1%), NaCl (150 mM), Tris-HCl (50 mM) and Halt™ protease and phosphatase inhibitor cocktail (Thermo Fisher Scientific, Waltham, MA, USA)]. The cell lysates were divided into three portions. One portion was stored at −20 °C and used as a lysate sample, one portion was incubated with control agarose beads conjugated with IgG antibody, and one portion was incubated with agarose beads conjugated with anti-Flag antibody. After an overnight incubation at 4 °C with rotation, the beads were washed with 1 X Lysis buffer and suspended in 2 X Sample buffer before denaturing at 95 °C for 5 min. The immunoprecipitated proteins were separated by 8% SDS-PAGE, transferred to a nitrocellulose membrane and blocked with 5% non-fat dry milk at room temperature for 1 h. The blocked membrane was then cut into strips and the strips were incubated with either anti-SVIL (Sigma-Aldrich, St. Louis, MO, USA, 1:1000 dilution) or anti-Flag (Sigma-Aldrich, St. Louis, MO, USA, 1:1000 dilution) antibody overnight at 4 °C. After washing with 1 X Tris-buffered saline containing 0.1% Tween 20, the membrane was incubated with an anti-mouse or anti-rabbit secondary antibody (Cell signaling, Danvers, MA, USA, 1:2000 dilution) for 1 h at room temperature, followed by washing and development with SuperSignal West Pico Chemiluminescent Substrate (Thermo Scientific, Waltham, MA, USA).

### 2.9. Western Blot Assay

hOAS1 proteins expressed in bacteria or in mammalian cells were separated on a 10% SDS-PAGE gel and transferred to a nitrocellulose membrane at 100 V for 1 h. The membrane was incubated in blocking buffer (1 X Tris-buffered saline containing 5% non-fat dry milk and 0.1% Tween 20) at room temperature for 1 h and then cut into strips. To detect the hOAS1 isoform proteins expressed in bacteria, the membrane strips were incubated with anti-V5 (Sigma-Aldrich, St. Louis, MO, USA; 1:1000) antibody at 4 °C overnight. To detect overexpressed hOAS1 isoforms in HEK293 cell lysates, the membrane strips were incubated at 4 °C overnight with mouse anti-Flag antibody (Sigma-Aldrich, St. Louis, MO, USA; 1:1000) or mouse anti-β actin antibody (Cell signaling, Danvers, MA, USA; 1: 10,000). The membranes were washed three times for 10 min with 1 X Tris-buffered saline containing 0.1% Tween 20, and then incubated with a horseradish peroxidase-conjugated secondary antibody (Santa Cruz Biotechnology, Dallas, TX, USA) for 1 h at room temperature. After washing, the membrane strips were processed for enhanced chemiluminescence using a Super-Signal West Pico detection kit (Thermo Fisher Scientific, Waltham, MA, USA) according to the manufacturer’s protocol.

## 3. Results 

### 3.1. In Vitro Expression of Recombinant hOAS1 Isoforms

**Figure 1:**
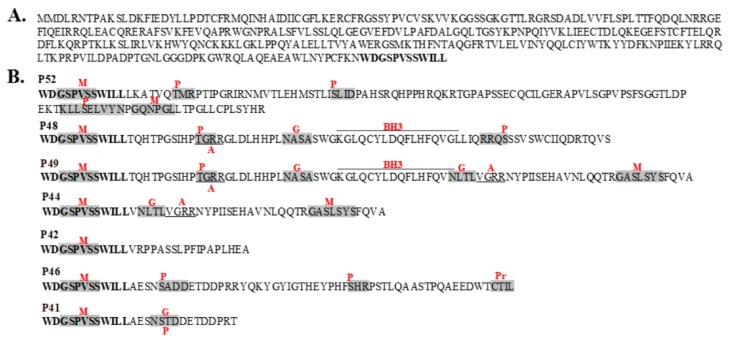
The unique C-terminal amino acid sequences of seven hOAS1 isoform proteins. (**A**) The common N-terminal sequence shared by all of the hOAS1 isoforms. The bolded C terminal sequence is repeated in the bottom panel. (**B**) The unique C-terminal sequences of the isoforms. Predicted post-translational modification sites or domains are indicated by red letters. M, N-myristoylation site; P, various predicted phosphorylation sites; G, N-linked glycosylation site; A, amidation site; Pr, prenylation site; BH3, BH3 domain. Post translational modification sites were predicted using PROSITE (https://prosite.expasy.org).

Seven isoforms of hOAS1, p41, p42, p44, p46, p48, p49 and p52, were detected after amplification and cloning of hOAS1 cDNAs from cellular RNA extracted from IFNβ-treated human CCL-110 or CCL-66 fibroblasts. The OAS1 isoforms differed only in their C-terminal sequences (Figure 1). Predicted post translational modifications on the unique C-terminal regions of each isoform protein are indicated.

**Figure 2:**
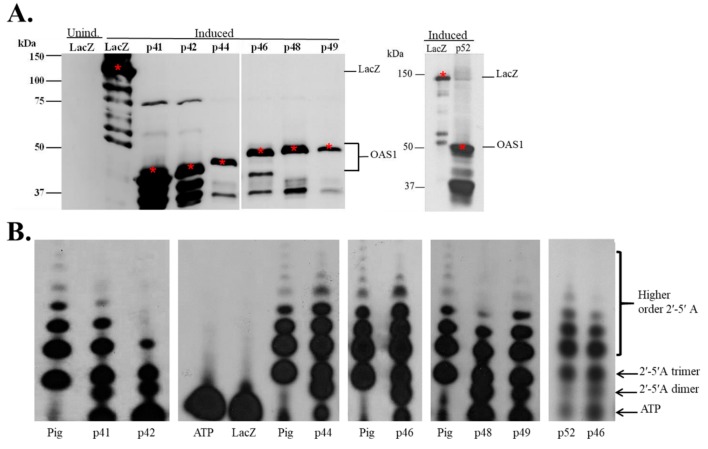
Bacterial expression of hOAS1 isoform proteins and analysis of their ability to produce 2-5A. (**A**) Seven hOAS1 isoform fusion proteins and LacZ protein were expressed individually in bacteria. After isopropyl β-d-1-thiogalacto-pyranoside (IPTG) (1 mM) induction, bacteria were lysed and proteins in the crude bacterial lysates were separated by 10% sodium dodecyl sulfate-polyacrylamide gel electrophoresis (SDS-PAGE), transferred to a nitrocellulose membrane and immunoblotted with anti-V5 antibody. All of the OAS1 isoforms except for p52 were run on the same gel. A lysate from uninduced bacteria transformed with a plasmid containing the LacZ gene was used as a negative control. The full-length proteins are indicated by a red asterisk. OAS1 degradation bands were detected on the gel below the full-length proteins. (**B**) After protein purification on a Talon metal affinity column, each human OAS1 isoform (22 μL) was incubated with α^32^P-adenosine triphosphate (ATP) and poly(I:C) for 18 h at 30 °C. LacZ protein plus α^32^P-ATP (Lane 5) and α^32^P-ATP alone (no protein) (Lane 4) were used as negative controls. Purified *Escherichia coli* (*E. coli*)-expressed pig OAS1 (0.5 μg) was used as a positive control. Two µL of each reaction were electrophoresed on a 20% polyacrylamide/8M urea denaturing gel and radiolabeled 2-5A was visualized by autoradiography. Separate gels used to analyze the 2-5A are indicated by spaces.

Each of the isoform proteins with N-terminal 6 X His and V5 fusion tags was expressed in *E. coli*. Recombinant LacZ protein was expressed from the same vector with the same N-terminal tags and used as a negative control. Although clear induction of the hOAS1 fusion protein expression could not be detected by Coomassie blue staining following 10% SDS-PAGE of the bacterial extracts, proteins of the expected molecular masses were detected in the induced bacterial lysates by Western blotting with anti-V5 antibody, confirming the expression of these fusion proteins (Figure 2A). Some breakdown of each of the hOAS1 isoforms was indicated by the detection of bands corresponding to N-terminal fragments. 

Some breakdown was also observed for the LacZ control protein, which was expressed from the same plasmid and in the same cells as the hOAS1 isoforms. The poor expression levels of the hOAS1 isoforms indicate that some cytotoxicity was associated with their expression in bacteria. This finding is consistent with previously published data obtained for murine Oas1 fusion proteins expressed in bacteria [19]. The hOAS1 isoforms and recombinant LacZ were partially purified on Talon metal affinity columns.

### 3.2. Recombinant hOAS1 p41, p42, p44, p46, p48, p49 and p52 Isoforms Are Functionally Active

To assess the 2-5A synthetase activity of the hOAS1 isoforms, each partially purified isoform was incubated with poly(I:C) and α^32^P-ATP at 30 °C for 18 h. The 2-5A produced was separated on a denaturing 20% PAGE/8M urea gel and visualized by autoradiography (Figure 2B). Lane 4 contained only α^32^P-ATP and revealed the position of free ATP. LacZ did not produce any 2-5A (Lane 5). 

An *E. coli*-expressed, purified pig OAS1 protein (a gift from Rune Hartmann, University of Aarhus, Denmark) was used as a positive control for 2-5A synthesis [20]. This protein is 73% identical in sequence to the human p46 isoform, and was previously shown to have similar synthetase activity [20].

The hOAS1 p41, p42, p44, p46, p48, p49 and p52 isoforms synthesized 2-5A dimers, trimers and higher order 2-5As, in response to poly(I:C) activation, indicating that each isoform is functionally active, and that the presence of the N-terminal 6 X His and V5 tags did not inhibit synthetase activity. P42 synthetized tetramers, but did not efficiently synthesize higher order 2-5A. The Pig OAS1 produced few dimers compared to the hOAS1 isomers under the 18 h reaction incubation conditions used. The p42, p44, p46, p48 and p52 hOAS1 proteins were previously shown to have 2-5A synthetase activity [14,21,22]. Consistent with our data, a recent study that directly compared the relative activities of partially purified p42, p44, p46, p48 and p52 expressed in *E. coli* as maltose-binding protein (MBP) fusion proteins, found that all of the isoforms were able to robustly synthesize 2-5A [23]. However, at low concentrations, the p44 and p52 isoforms had lower activities than the other isoforms. The concentrations of full-length active protein varied among the isoforms in our study, and the less efficient activity observed for p42 may have been due to a lower amount of active enzyme in the assay. Our data, as well as that of others, indicate that the variable C-terminal sequences of the different isoforms have little effect on their ability to synthesize 2-5A.

### 3.3. The hOAS1 p42, p44, p46, p48 and p52 Isoforms Are Functionally Active in Mammalian Cells

**Figure 3:**
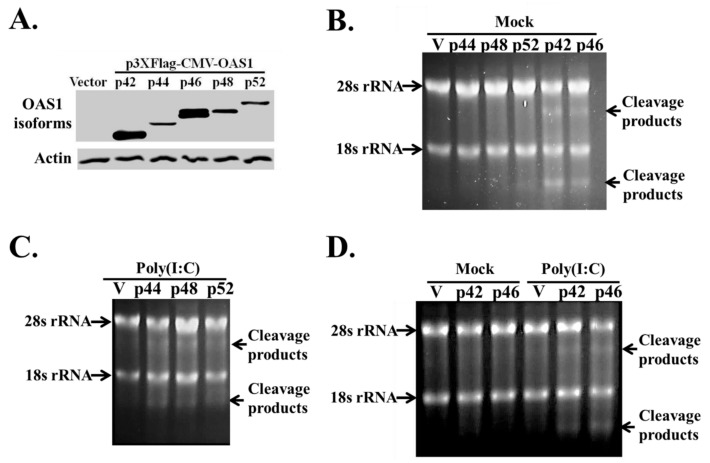
Analysis of RNase L cleavage in cells overexpressing individual hOAS1 isoforms in response to poly(I:C). HEK293 cells were transfected with 1 µg of empty vector DNA, or of one of the hOAS1 isoforms cloned into the p3xFlag-CMV plasmid for 24 h. (**A**) Western blot analysis of the expression level of each OAS1 isoform, using anti-Flag and anti-β actin antibodies. HEK293 cells overexpressing each OAS1 isoform (1 µg of plasmid DNA) for 24 h were either mock transfected (**B**), or transfected with poly(I:C) for 6 h (**C**). Total intracellular RNA was extracted and separated on a denaturing agarose gel and stained with ethidium bromide. (**D**) HEK293 cells were transfected with 0.5 µg p42 or p46 plasmid DNA for 9 h, followed by either mock transfection, or transfection with poly(I:C) for 6 h. Total intracellular RNA was extracted and separated on a denaturing agarose gel and stained with ethidium bromide. The 18S/28S rRNA and their cleavage products are indicated by arrows. V: empty vector control.

A previous study in human A549 cells showed that a Dengue virus infection activated RNase L cleavage in cells overexpressing either the p42 or p46 hOAS1 isoforms, but not the p44, p48 or p52 isoforms [18]. To test whether or not each of these five hOAS1 isoforms can be activated by poly(I:C) when expressed in mammalian cells, the p42, p44, p46, p48 and p52 isoforms were subcloned into the p3xFlag-CMV mammalian expression vector with a 3 X Flag tag fused at the N-terminus, and then individually overexpressed in HEK293 cells, which exhibit low endogenous hOAS1 basal activity in response to poly(I:C). HEK293 cells were lysed at 24 h after transfection with an individual hOAS1 isoform cDNA (1 µg of plasmid DNA), and isoform expression levels were analyzed by Western blotting using anti-Flag antibody. The p42 and p46 isoforms were expressed at much higher levels than the p44, p48 and p52 isoforms (Figure 3A). It was previously shown that hOAS1 p42 or p46 proteins were expressed in yeast at higher levels than p44, p48 or p52 [23]. In another study, although the transcript levels for Xpress tagged p42, p44, p46 and p48 were equivalent in HEK 293T cells after 48 h of expression, p44 and p48 had impaired protein expression compared to p42 and p46. Also, only endogenous p42, p46 and p48 were detected at the protein level in non-transfected cells [24]. To analyze isoform-specific synthetase activity, HEK293 cells were transfected with an hOAS1 isoform cDNA (1 µg of plasmid DNA) and 24 h later, cells were either mock- or poly(I:C)-transfected for 6 h. Total intracellular RNA was then extracted and separated on a denaturing agarose gel to detect cellular rRNA cleavage by activated RNase L. Surprisingly, the most efficiently expressed isoforms (p42 and p46) both activated RNase L cleavage, as indicated by the additional bands detected below the 18S and 28S rRNA bands in the absence of poly(I:C) transfection (Figure 3B). In contrast, the overexpressed p44, p48 and p52 isoforms only activated RNase L cleavage following poly(I:C) transfection (Figure 3C). HEK293 cells were next transfected with 0.5 µg instead of 1 µg of p42 or p46 isoform cDNA containing plasmid DNA for 9 h instead of 24 h, and then mock- or poly(I:C)-transfected for 6 h prior to total cellular RNA extraction. Under these conditions, both p42 and p46 required poly(I:C) stimulation to activate detectable RNase L cleavage of rRNA (Figure 3D). The data indicate that the hOAS1 p42, p44, p46, p48 and p52 isoforms can be activated by poly(I:C) and stimulate RNase L activity, as detected by rRNA cleavage in HEK293 cells. The lower levels of RNase L activation by the p44, p48 and p52 isoforms appears to be due to the decreased levels of these proteins. Similarly, the robust activation of RNase L in yeast by hOAS1 p42 and p46 was attributed to the higher levels of these proteins [23].

### 3.4. Identification of Novel Binding Partners for hOAS1 p42 and p44 Isoforms in a Yeast Two-Hybrid Assay

A few examples of murine and human OAS1 family members interacting with a cellular protein to carry out novel functions independent of the OAS-RNase L pathway have been reported [16,25,26]. The different C-terminal sequences of the hOAS1 isoforms may mediate interactions with different binding partners. The hOAS1 p42 and p44 isoforms are predicted to be expressed by humans of all genotypes [11,12]. A yeast two-hybrid screen of a universal human cDNA library was performed to identify novel candidate peptide partners for these two isoforms. Full-length hOAS1 p42 or p44 were expressed as the bait. After screening approximately 2 × 10^6^ clones, five and three peptide candidates were identified as putative binding partners for p42 and p44, respectively (Table 1). Two overlapping peptides were identified for FBN1.

A yeast co-transformation assay was next used to further test binding between a hOAS1 isoform and each of the peptide candidates. The yeast cells transformed with a hOAS1 isoform and an identified prey peptide were cultured under three different levels of stringency: Under the double drop out condition (DDO), Trp and Leu are deleted from the media; under the triple drop out condition (TDO), His, Trp and Leu are deleted, and under the quadruple drop out (QDO) condition, His, Trp, Leu and Ade are deleted. In the yeast two hybrid system used, growth of yeast colonies on DDO plates indicated successful transformation of both a hOAS1 isoform and a peptide candidate in the same cell. The expression of His is controlled by the G1 promoter, and the expression of Ade is controlled by the G2 promoter. The sequences of these two promoters are unrelated except for the short protein binding sites that specifically bind to the Gal4 DNA binding domain linked to the bait. Therefore, for the transformed yeast to be able to grow on QDO plates, both the G1 and G2 promoters must be activated by the interaction of the prey and bait. To further increase the stringency, Aureobasidin A was added to the TDO (TDO/A) and QDO (QDO/A) media. Only yeast containing interacting prey and bait will express the AUR1-C gene regulated by the M1 promoter, which confers strong resistance to the otherwise highly toxic drug Aureobasidin A. Growth of yeast colonies on TDO/A and QDO/A plates indicated that the transformed hOAS1 isoform and peptide candidate interacted with each other in yeast. SV40 large T-antigen and murine p53 are known to interact, and were co-transformed as a positive control. SV40 large T-antigen and Lamin have not been reported to interact, and were co-transformed as a negative control. As an additional negative control, empty vector DNA was separately co-transformed with each of the screened peptide candidates. The peptide candidates, Fibrillin1 (FBN1) and Supervillin (SVIL) were identified as binding partners for hOAS1 p42 and p44, respectively, by the co-transformation assay (Table 2). An interaction between the p42 and voltage-dependent anion channel 2 (VDAC2) peptides was indicated by growth of colonies on the TDO/A plates, but QDO/A colonies were not detected. Cell growth on both the TDO/A and QDO/A plates was detected for the phosphoglucomutase 1 (PGM1) peptide after co-transfection with either empty vector DNA or cDNA encoding p42, indicating that this interaction was a false-positive.

### 3.5. Interaction of In Vitro Synthesized hOAS1 p42 and p44 Isoform Proteins and Their Identified Binding Partners

**Figure 4:**
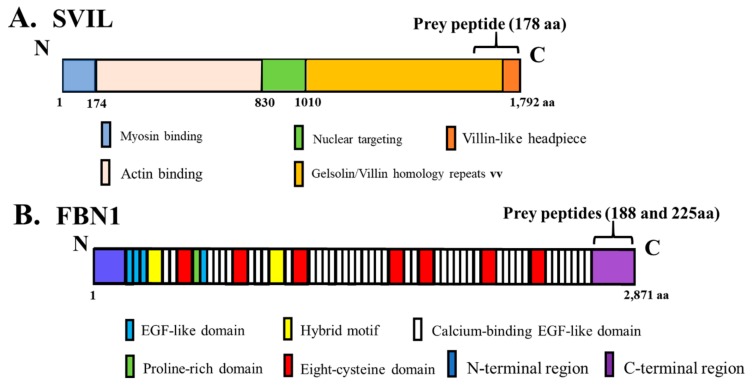
(**A**) Diagram of the domain structure of the full-length SVIL protein. The domains are color-coded, and the location of the 178 aa prey peptide (aa 1615–1792) is indicated by a bracket. The amino acid numbers are indicated below the diagram. (**B**) Diagram of the domain structure of the full-length FBN1 protein. The domains are color-coded, and the location of the two overlapping prey peptides, 188 aa (aa 2684–2871) and 225 aa (aa 2647–2871), are indicated by a bracket. EGF: epidermal growth factor.

**Figure 5:**
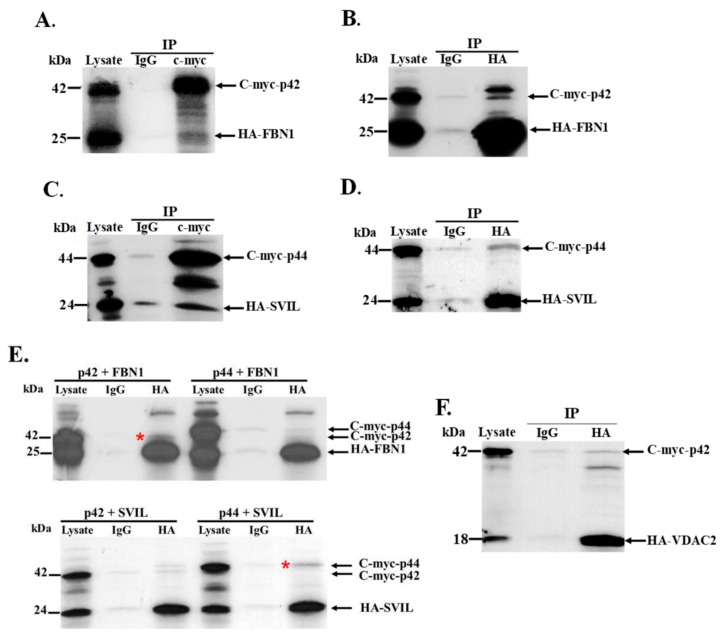
Analysis of the interaction between human OAS1 p42 and p44 isoforms and their respective binding partners in vitro. Wheat germ extract was used for the in vitro transcription and translation of c-myc tagged full length human OAS1 isoforms and of HA-tagged FBN1 (188 aa), SVIL or voltage-dependent anion channel (VDAC) peptides in the presence of [^35^S]-methionine. Reciprocal pull-down assays were performed with anti-HA and anti-c-myc antibodies. Non-specific IgG was used as the negative control. The protein complexes were resolved by SDS-PAGE and detected by autoradiography. (**A**) HA-FBN1 peptide and full-length c-myc-OAS1 p42 immunoprecipitated with anti-c-myc antibody. (**B**) C-myc-OAS1 p42 and HA-FBN1 peptide immunoprecipitated with anti-HA antibody. (**C**) HA-SVIL peptide and c-myc-OAS1 p44 immunoprecipitated with anti-c-myc antibody. (**D**) C-myc-OAS1 p44 and HA-SVIL peptide immunoprecipitated with anti-HA antibody (**E**) (top panel) Either C-myc-OAS1 p42 or p44 and HA-FBN1 peptide immunoprecipitated with anti-HA antibody and (bottom panel) either C-myc-OAS1 p42 or p44 and HA-SVIL peptide immunoprecipitated with anti-HA antibody. The hOAS1 isoform specifically immunoprecipitated is indicated with a red asterisk. (**F**) C-myc-OAS1 p42 and HA-VDAC2 peptide immunoprecipitated with anti-HA antibody. IgG: non-specific antibody control; IP: immunoprecipitation.

The yeast co-transformation assay detected binding between an FBN1 peptide and p42, as well as between an SVIL peptide and p44. The domains of these two partner proteins and the locations of the OAS1-interacting peptides within these proteins are shown in Figure 4. The data also suggested possible binding between VDAC2 and p42 in yeast. However, it was possible that these interactions might be indirect and/or mediated by another protein in yeast. To test whether or not p42 and p44 interact directly with the identified binding partner peptides, a wheat germ extract was used for in vitro transcription and translation of each of the c-myc-tagged full-length hOAS1 isoforms and of the HA-tagged peptide partners in the presence of [^35^S]-labeled methionine. After incubation of each hOAS1 isoform with a single binding partner peptide, in vitro immunoprecipitation (IP) of p42 or p44 was performed using anti-c-myc antibody. Increased co-precipitation of FBN1 by p42 and of SVIL by p44 compared to the non-specific IgG control was observed (Figure 5A,C). In reciprocal immunoprecipitation experiments using anti-HA antibody, the FBN1 and SVIL peptides also co-precipitated with p42 and p44, respectively, indicating a direct interaction in vitro (Figure 5B,D). To further analyze the specificity of these interactions, co-immunoprecipitation assays were performed with the FBN1 peptide and either p42 or p44, and also with the SVIL peptide and either p42 and p44 (Figure 5E). Only p42 was enriched in the FBN1 assay, and only p44 was enriched in the SVIL assay. In contrast, the amount of VDAC2 peptide was not enriched over the amount in the nonspecific control IgG sample after immunoprecipitation of p42 (Figure 5F), indicating that a direct interaction between p42 and the VDAC2 peptide did not occur under in vitro conditions. The faint co-IP bands observed in the in vitro TnT assays may be due to the lack of translational modifications and/or suboptimal peptide folding.

### 3.6. The hOAS1 p44 Isoform Interacts with Endogenous SVIL Protein in Mammalian Cells

**Figure 6:**
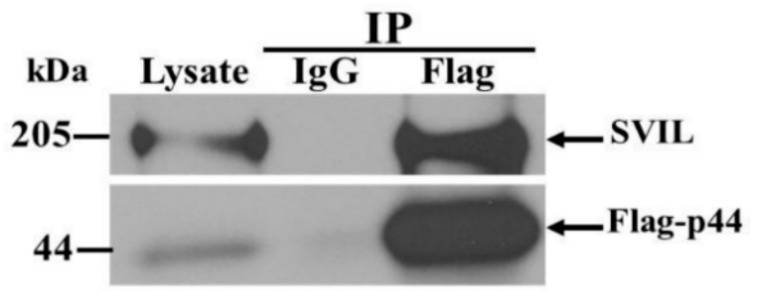
Interaction between the hOAS1 p44 isoform and endogenous full-length SVIL in A549 cell extracts. Human A549 cells were transiently transfected with plasmid DNA expressing the 3xFlag-tagged full-length hOAS1 p44 isoform. At 48 h post transfection, cell lysates were collected and incubated with anti-Flag antibody-conjugated agarose beads. Non-specific IgG conjugated agarose beads were used as the negative control. After co-immunoprecipitation, protein complexes were resolved by SDS-PAGE, transferred to a nitrocellulose membrane and detected by anti-Flag and anti-SVIL antibodies.

As a further analysis of the protein–protein interactions detected, the interaction of a full length hOAS1 isoform protein with its full-length endogenous protein partner was tested. A549 cells were transfected with a cDNA expressing a FLAG-tagged p44. Cell extracts were harvested at 48 h after transfection, and immunoprecipitated with anti-Flag antibody. The immunoprecipitated complex was separated by SDS-PAGE and probed by Western blotting using anti-Flag and anti-SVIL antibodies (Figure 6). 

Compared to the non-specific IgG control and the lysate input, enrichment of endogenous full-length SVIL was detected in complexes precipitated with anti-Flag antibody. These data support an interaction between the hOAS1 p44 isoform and the endogenous full-length SVIL protein in human cells.

## 4. Discussion

The OAS/RNase L system is an important component of the IFN-dependent antiviral response in cells [27]. The human genome encodes a single OAS1 gene, but multiple isoform proteins are produced due to alternative splicing, as well as to single nucleotide variations that affect splicing [6,11,12,13,14,15,16]. The hOAS1 isoforms differ in their C-terminal sequences. The crystal structures of the porcine OAS1 either alone or bound to dsRNA and of hOAS1 p46 indicate that the N- and C-terminal regions of these proteins interact [20,28,29]. The presence of alternative C-terminal ends in the different isoform proteins could affect protein folding, protein stability, synthetase activity, protein post-translational modification and/or protein intracellular localization.

When activated by dsRNA, OAS proteins produce 2-5A that induces dimerization and activation of host RNase L, that in turn cleaves both viral and cellular ssRNAs. In the present study, we showed that each of the seven bacterially-expressed hOAS1 isoform proteins tested could synthesize trimers and some higher order 2-5As in vitro, including p41 and p49. We also showed that poly(I:C) stimulation of hOAS1 p42, p44, p46, p48 or p52 expressed in HEK293 cells activated RNase L cleavage of cellular 18S/28S rRNA, indicating that each of these isoforms can be activated by poly(I:C) in mammalian cells. The data indicate that the alternate C-terminal sequences do not negatively affect 2-5A synthetase activity.

Antiviral activity has been previously reported to be associated with the expression of specific hOAS1 isoforms in cells. An A-allele in the intron-5/exon-6 splice acceptor site that leads to the expression of p48 and p52, but not p46, was reported to correlate with increased risk of WNV infections in humans [30]. Another study reported that Dengue virus replication is blocked by an RNase L-dependent mechanism in human cells overexpressing p42 or p46, but not p44, p48 or p52 [18]. In the present study, comparison of the expression efficiencies of different hOAS1 isoforms indicated that the p42 and p46 isoforms were expressed at much higher levels in HEK293 cells than the other isoforms tested, suggesting that these two isoforms may have a higher stability in a mammalian cell. A recent study also showed that p44 and p48 were expressed at lower levels compared to p42 and p46 after transfection of HEK 293 cells, even though the mRNA levels for all of the isoforms were similar [24]. Sequential truncation of the C-terminal ends of p44 and p48 resulted in increased expression levels, indicating that the C-terminal regions are responsible for the decreased stability of these two isoforms.

Surprisingly, the high expression levels of the p42 and p46 isoforms resulted in the activation of RNase L cleavage of 18S/28S rRNA in the absence of poly(I:C) treatment. Interestingly, using less DNA for transfection and a shorter transfection time abrogated the poly(I:C)-independent activation of rRNA cleavage. 

Although we found that each of the expressed isoforms was activated by transfection of poly(I:C), it is possible that the higher expression levels of the p42 and p46 isoforms in mammalian cells were the reason for the previous detection of RNase L activation by a Dengue virus infection only in cells overexpressing these two isoforms [18]. However, a recent study showed that hOAS3 was the primary hOAS protein activated by infection with another flavivirus Zika virus [31]. The means by which the hOAS1 p42 and p46 isoform proteins were activated to produce 2-5A after expression in HEK293 cells at high levels in the absence of poly(I:C), is not known. The conformational change induced by dsRNA has been shown to be closely linked to the catalytic activity of hOAS proteins [11,29]. Although, it was previously reported that in vitro-synthesized p42 and p48 require tetramerization for their activity via a C-F-K motif that is conserved in all OAS1 isoforms [32], it was subsequently demonstrated that monomeric p42 expressed in insect cells was fully active [11]. It is possible that concentration-dependent multimerization may have increased the sensitivity of p42 and p46 to endogenous dsRNA, and that this was responsible for the dsRNA-independent rRNA cleavage that we observed.

Although the majority of the predicted post-translational modifications of the C-terminal sequences of the various hOAS1 isoforms (Figure 1) would not be expected to be added by bacteria, these post translational modifications may differentially affect the stability of the various hOAS1 isoforms in mammalian cells. No post translational modification sites were predicted for the C-terminal region of p42, but multiple post translational modification sites were predicted for the p46 C terminal region, and p46 was previously predicted to associate with mitochondria [33]. The different C-terminal sequences and post translational modifications of the individual hOAS1 isoforms may mediate hOAS1 isoform interactions with unique protein partners that facilitate novel functions and/or determine intracellular localization. Previous studies have identified novel binding partners for some of the hOAS1 isoform proteins. The intracellular domain of the prolactin receptor was identified as a binding partner of the p42 isoform, and this interaction was suggested to modulate prolactin-induced STAT1 binding at gene promoters regulated by interferon-regulatory factor 1 [26]. The p48 isoform was predicted to contain a BH3 domain and to mediate cellular apoptosis by interacting with Bcl-2 and Bcl-X_L_ [16]. The hOAS1 p46 isoform was shown to associate with mitochondria [33]. The identification of these novel interactions suggested that individual hOAS1 isoforms are likely to have additional functions beyond a role in the canonical OAS-RNase L pathway. It is possible that the isoform proteins that are present in low levels could still be functionally important.

In the present study, SVIL was identified as a binding partner for the hOAS1 p44 isoform, and FBN1 was identified as a putative binding partner for the hOAS1 p42 isoform. However, the prolactin receptor was not reidentified as a p42 binding partner in our yeast two hybrid screen. Additional IP assays with an anti-FBN1 antibody are needed to further test the interaction of p42 with endogenous FBN1. FBN1 is an extracellular matrix glycoprotein that can downregulate the transforming growth factor beta (TGF-β) signaling pathway [34,35,36]. Previous publications have reported OAS activity in the serum, and showed that extracellular OAS1 could enter cells and exert an antiviral activity independent of RNase L [37,38,39]. It is possible that FBN1 could function as a scaffold protein to tether extracellular OAS1 proteins on the cell surface. RNase L was recently reported to bind to actin-binding protein filamin A, and to function as an innate immune component maintaining a cellular barrier to viral entry [40]. SVIL, the partner identified for p44, is an actin binding protein from the Villin family. It is possible that interaction between the hOAS1 p44 isoform and actin-bound SVIL could bring this isoform in close proximity to RNase L at the cytoskeleton barrier. The C-terminal headpiece domain of SVIL, which is located at the C-terminus of the identified peptide that interacts with p44, is conserved among many members in the Villin family [41,42], suggesting the possibility that the hOAS1 p44 isoform might also interact with other members of this family.

In summary we show that the alternative C-terminal sequences of the hOAS1 isoforms have little effect on activation by poly(I:C) or on the ability to synthesize 2-5A. The identification of novel binding partners for some of the hOAS1 isoforms in this and previous studies suggests that the different C-terminal sequences of the different isoform proteins may mediate differential protein–protein interactions that are required for cellular localization and possible alternative functions of the different isoform proteins.

## Figures and Tables

**Table 1:** Identification of peptide binding partners for the full-length hOAS1 p42 and p44 isoforms using a yeast two hybrid assay.

hOAS1 Bait	Peptide Candidate Size (aa)	Putative Protein Containing the Peptide
p42	188, 225	Fibrillin 1 (FBN1)
	95	Phosphoglucomutase 1 (PGM1)
	127	Voltage-dependent anion channel 2 (VDAC2)
	120	Trafficking protein particle complex 8 (TRAPPC8)
	115	Lysyl Oxidase-Like 3 (LOXL3)
p44	178	Supervillin (SVIL)
	206	G elongation factor, mitochondrial 2
	139	COX11 cytochrome c oxidase assembly homolog

**Table 2:** Confirmation of the peptide candidates as binding partners for the full-length human OAS1 p42 and p44 isoforms using a yeast co-transformation assay.

Peptide Candidate	Co-Transformed Bait	DDO ^a^	TDO/A ^a^	QDO/A ^a^
FBN1	p42	>100 ^b^	7 ^b^	1 ^b^
	Empty vector	>100	-	-
PGM1	p42	>100	8	1
	Empty vector	>100	2	2
VDAC2	p42	>100	17	-
	Empty vector	>100	-	-
KIAA1012	p42	>100	-	-
	Empty vector	>100	-	-
LOXL3	p44	>100	-	-
	Empty vector	>100	-	-
SVIL	p44	>100	6	1
	Empty vector	>100	-	-
G elongation factor	p44	>100	-	-
	Empty vector	>100	-	-
COX11	p44	>100	-	-
	Empty vector	>100	-	-
T antigen	p53 ^c^	>100	43	14
T antigen	Lambda ^d^	28	-	-

^a^ Growth conditions with different stringencies. DDO: minus Trp and Leu; TDO/A: minus His, Trp and Leu and supplemented with Aureobasidin A; QDO/A: minus Ade, His, Trp and Leu and supplemented with Aureobasidin A. ^b^ Number of yeast colonies that grew on the plates. ^c^ Positive control. ^d^ Negative control.
